# Combined effects of GSTP1 and MRP1 in melanoma drug resistance

**DOI:** 10.1038/sj.bjc.6602681

**Published:** 2005-07-05

**Authors:** P Depeille, P Cuq, I Passagne, A Evrard, L Vian

**Affiliations:** 1 Laboratoire de Toxicologie du Médicament – EA 2994, Faculté de Pharmacie, BP 14491, 15 avenue Charles Flahault, 34093 Montpellier cedex 5, France

**Keywords:** glutathione-S-transferase, multidrug resistance protein, antisense RNA, etoposide, melanoma

## Abstract

Glutathione-S-transferase Pi1 (GSTP1) and multidrug resistance protein 1 (MRP1) are overexpressed in melanoma, a skin cancer notoriously resistant to all current modalities of cancer therapy. To investigate the involvement of these detoxifying enzymes in the drug resistance of melanoma, an inducible (Tet-On™ system) antisense (AS) RNA strategy was used to specifically inhibit GSTP1 expression in A375 cells, a human melanoma cell line expressing high levels of GSTP1 and MRP1. Stable transfectant clones were established and analysed for GSTP1 inhibition by AS RNA. The clone A375-ASPi1, presenting a specific 40% inhibition of GSTP1 expression in the presence of doxycycline, was selected. Lowering the GSTP1 level significantly increased (about 3.3-fold) the sensitivity of A375-ASPi1 cells to etoposide. Inhibitors of glutathione synthesis (BSO), GSTs (curcumin, ethacrynic acid), and also of MRPs (MK571, sulphinpyrazone) improved the sensitising effect of GSTP1 AS RNA. All these inhibitors had stronger sensitising effects in control cells expressing high GSTP1 level (A375-ASPi1 cells in the absence of doxycycline). In conclusion, GSTP1 can act in a combined fashion with MRP1 to protect melanoma cells from toxic effects of etoposide.

Development of drug resistance is a key element in the failure of chemotherapy treatment. Melanoma, the most aggressive form of skin cancer, is notoriously resistant to all current modalities of cancer therapy (Helmbach *et al*, 2001). A large set of genetic, functional and biochemical studies suggest that melanoma cells become insensitive to a variety of chemotherapeutic drugs by exploiting their intrinsic resistance to apoptosis and by reprogramming their proliferation and survival pathways during melanoma progression. Among the systems potentially involved in melanoma cell chemoresistance, the overexpression of glutathione S-transferases (GSTs) and multidrug resistance proteins (MRPs) may play an important role (Schadendorf *et al*, 1995b; Moral *et al*, 1997; Serrone and Hersey, 1999).

Glutathione-S-transferases are a family of phase II detoxification enzymes that catalyse the conjugation of glutathione (GSH) to a wide variety of electrophilic compounds, including anticancer drugs but also carcinogens or mutagens (Tew, 1994; Townsend and Tew, 2003). Glutathione-S-transferases are divided into two distinct super-families: the membrane-bound microsomal and the cytosolic GSTs (Townsend and Tew, 2003). Human cytosolic GSTs are highly polymorphic and can be divided into six classes (namely alpha, Mu, Pi, Theta, Omega and Zeta) (Townsend and Tew, 2003).

The alpha, Pi and Mu classes of GSTs are the most studied and the most implicated in cellular resistance (Tew, 1994). Thus, tumours (Schisselbauer *et al*, 1990) or cell lines (Tew, 1994; Hayes and Pulford, 1995) show an increased GST level after development of drug resistance, cancer cells transfected with GSTs can express a drug resistance (Moscow *et al*, 1989; Manoharan *et al*, 1991; Morrow *et al*, 1998; Depeille *et al*, 2004) and the inhibition by antisense (AS) gene of endogenous GSTP expression reduces drug resistance (Ban *et al*, 1996). The first mechanism by which GSTs can confer drug resistance is direct detoxification. Usually, electrophiles are made less reactive by conjugation with glutathione, and the conjugates are thought to be less toxic to the cell. More recently, a plausible second role of GSTs in the development of drug resistance through the inhibition of the mitogen-activated protein (MAP) kinase pathway by protein: protein interactions was evoked (Adler *et al*, 1999; Townsend and Tew, 2003). However, some other investigators have not found associations between cellular resistance to anticancer agents and expression of GSTs (Townsend *et al*, 1992). Thus, the role of GSTs in the protection of cells against anticancer drugs remains equivocal.

The overexpression of GSTs is not always sufficient to confer significant protection from the electrophiles. Thus, it has been shown that GSTs must be coexpressed with MRPs, particularly MRP1 and MRP2, to protect cells from anticancer agents (Morrow *et al*, 1998; Harbottle *et al*, 2001; Depeille *et al*, 2004; Smitherman *et al*, 2004). Multidrug resistance protein 1 and MRP2 belong to the nine members family of ABC transporters, discovered by Cole *et al* (1992), that are responsible for the active transport across biological membrane of structurally diverse lipophilic anions (Borst *et al*, 1999; Kruh and Belinsky, 2003). Multidrug resistance protein 1, MRP2 and MRP3 have overlapping substrate specificity, including glutathione-conjugates (Keppler *et al*, 2000). Multidrug resistance protein 4 an MRP5 are involved in nucleotide efflux (Wijnholds *et al*, 2000). Initially, MRP1 and MRP2 have been shown to confer resistance to various drugs of natural origin, including anthracyclines, vinca alkaloids and epipodophyllotoxins (Borst *et al*, 1999). Then, the requirement of GSH for MRP1-mediated cellular efflux of some natural products was demonstrated (Rappa *et al*, 1997). Finally, it was reported that detoxification of anticancer agents implicated a combined action of GSTs and MRPs (Morrow *et al*, 1998; O'Brien *et al*, 2000; Harbottle *et al*, 2001; Depeille *et al*, 2004).

Human melanoma expresses high levels of GSTP1 (Moral *et al*, 1997) and MRP1 (Schadendorf *et al*, 1995b). The aim of this study was to investigate the role of GSTP1 and MRPs in resistance of this skin tumour to drugs. To address this question, an inducible AS RNA strategy we used to, in a tetracycline-controlled fashion, specifically inhibit GSTP1 expression in the GSTP1 and MRP1 expressing A375 human melanoma cells. The efficacy of the GST inhibition (mRNA and protein expression, enzyme activity) by AS RNA was quantified in the cell lines. The effects of the lowering of GSTP1 expression level on drug sensitivity were assessed. Finally, GST and MRP inhibitors were used to confirm the results obtained using AS approach.

## MATERIALS AND METHODS

### Drugs and chemicals

Geneticin and hygromycin were from Life Technologies (Cergy Pontoise, France). [^3^H]-etoposide (0.654 Ci mmol^− 1^) was purchased from Moravek Biochemicals (Brea, CA, USA). Doxycycline was from Clontech (St Quentin Yvelines, France). MK571 was from Biomol International (Le Perray en Yvelines, France) (10 mM in H_2_O). All other drugs were from Sigma (St Quentin-Fallavier, France). Stock solutions were vincristine (100 *μ*M in H_2_O), doxorubicin (3.7 mM in H_2_O), mitomycin C (10 mM in H_2_O), chlorambucil (100 mM in ethanol), melphalan (100 mM in acidified ethanol), 1-chloro-2,4-dinitrobenzene (CDNB) (100 mM in PBS), glutathione (GSH) (100 mM in H_2_O), cisplatin (5 mM in 0.9% NaCl), mitoxantrone (5 mM in dimethyl sulphoxide), etoposide (5 mM in dimethyl sulphoxide), curcumin (2.5 mM ethanol), ethacrynic acid (2.5 mM in H_2_O), sulfinpyrazone (200 mM in dimethyl sulphoxide) and D,L-buthionine-[*S,R*]-sulphoximine (BSO) (5 mM in H_2_O).

The cDNA encoding on one hand human GSTA1, GSTP1 and GSTM1, on the other hand human MRP1 and MRP2 were kindly provided by Dr CS Morrow (Wake Forest University School of Medicine, Winston-Salem, USA) and Pr D Keppler (DKFZ, Heidelberg, Germany), respectively.

### Construction of a GSTP1 AS vector

The full-length cDNA encoding human GSTP1 was amplified by PCR using standard procedures and the following primers: ASPi-front 5′-GATATCGCGGCCGCATGCCGCCCTACACCGTGGTC-3′, and ASpi-reverse 5′-GGATCCACGCGTTCACTGTTTCCCGTTGCCATT-3′. Amplified sequence and pTRE2hyg (Clontech, St Quentin Yvelines, France) were digested by *Bam*HI and *Eco*RV (Eurogentec, Angers, France). Both of these processed fragments were then ligated with T4 ligase (Life Technologies, Cergy Pontoise, France), and a clone was selected in which the GSTP1 cDNA was inserted in the reverse direction. This clone was named ppTRE-ASPi.

### Cells lines

A375 human malignant melanoma (MM) cells (CRL-1619, American Type Culture Collection, Manassas, VA, USA) were grown at 37°C in a fully humidified 5% CO_2_ atmosphere in DMEM supplemented with 10% Tet-system approved foetal bovine serum (Clontech, St Quentin Yvelines, France). A375 cells expressing human GSTP1 inducible AS RNA were established by transfection (LipofectAMINE™, Invitrogen, Cergy Pontoise, France) of A375-wt cells with Tet-On™ gene expression system (Clontech, St Quentin Yvelines, France) consisting of two vectors. First, cells were transfected with pTet-On, expressing reverse tetracycline-controlled transactivator (rtTA), and clones were selected for geneticin (1 mg ml^− 1^) resistance and for rtTA expression level (luciferase assay, according to manufacturer's instruction). Then clones were transfected with ppTRE-ASPi, a pTRE2hyg vector containing the cDNA encoding the human GSTP1 AS RNA and selected for antibiotic resistance (1 mg ml^− 1^ geneticin and 0.4 mg ml^− 1^ hygromycin). One clone, namely A375-ASPi1, presenting a specific and significant inhibition of GSTP1 expression in the presence of doxycycline (0.2 *μ*g ml^− 1^, 24 h) was used for further analysis.

### Analysis of GST and MRP expression

#### Reverse transcription polymerase chain reaction

Extraction of total cellular RNA was carried out using Trizol™ reagent (Invitrogen, Cergy Pontoise, France) and expression was analysed by reverse transcription polymerase chain reaction (RT—PCR) using standard procedures and specific primers (Depeille *et al*, 2004). Positive controls were either plasmids containing the coding sequences for GSTs (A1, P1 and M1) and MRPs (1 and 2), or Caco-2 cells for MRP3 (Depeille *et al*, 2004). PCR products were run on agarose gels supplemented with ethidium bromide and visualised by ultraviolet illumination. Band intensities were quantified by densitometry analysis using Bio1D software (Vilber Lourmat, Marne La Vallée, France).

#### Quantitative real-time PCR

Extraction of total cellular RNA was carried out using RNA easy kit (Qiagen, Courtabœuf, France) according to the manufacturer's procedure. Reverse transcription was done using standard procedure. Glutathione-S-transferase P1 expression was determined by real-time PCR on a LightCycler instrument (Roche Diagnostics, Meylan, France) using a SYBRGreen® fluorescent dye, which binds to the double-stranded DNA yielding fluorescence. The cDNA amplication was performed in 40 cycles as follows: initial predenaturation 95°C / 10 min, denaturation 95°C / 10 s, annealing 58°C / 6 s, extention 72°C / 10 s. All the steps were performed in Faststart DNA Master SYBR Green I mix (Roche Diagnostics, Meylan, France) using specific primers (0.5 *μ*M) (Depeille *et al*, 2004) and 4 mM MgCl_2_. The specificity of amplication was checked using post-PCR melting curves analysis with Tm determination. During exponential phase of PCR reaction, the crossing threshold (*C*_T_) was determined for each amplification curves. CAL1-*μ*1 cell line (Depeille *et al*, 2004) was used as calibrator. All results were normalized with beta-actin gene and expressed as ratios related to GSTP1 expression in the calibrator. The relative quantification ratio was evaluated using quantification based on *C*_T_ method with an efficiency (*E*) correction: 
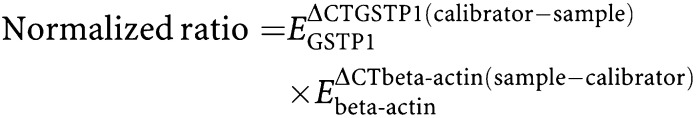


#### Western blot analysis

The expression of GSTs in A375 cells was investigated as previously described (Depeille *et al*, 2004). Briefly, cells treated or not with doxycycline (0.2 *μ*g ml^− 1^, 24 h) were homogeneized in lysis buffer. After centrifugation cytosolic proteins (20 *μ*g) were separated by 12% SDS–polyacrylamide gel electrophoresis and electroblotted onto nitrocellulose membrane. The membranes were blocked in 10% milk in PBS-Tween followed by incubation with rabbit polyclonal primary antibodies anti-GST alpha or mu and with mouse monoclonal primary antibody anti-GST Pi 1 : 500 (Novocastra-Tebu, Le Perray en Yvelines, France), or mouse monoclonal primary antiboby anti-beta-actin 1 : 2000 (Sigma, St Quentin Fallavier, France). Then, membranes were incubated with anti-species peroxydase-conjugated secondary antibodies 1 : 5000 (Sigma, St Quentin Fallavier, France) and visualised using enhanced chemiluminescence (ECL) reagents (Amersham Biosciences, Saclay, France). Band intensities were quantified by densitometry analysis using Bio1D software (Vilber Lourmat, Marne La Vallée, France).

To study MRP expression, cells were lysed on ice in RIPA buffer (20 mM Tris pH 7.5, 150 mM NaCl, 1% Triton X-100, 1% sodium desoxycholate, 0.1% SDS) supplemented with fresh 1 mM DTT, 2 mM NaF and protease inhibitor cocktail (Sigma) (Depeille *et al*, 2004). The lysates were centrifuged and the supernatants proteins (100 *μ*g) were separated by 7% SDS–polyacrylamide gel electrophoresis and electroblotted at 4°C onto nitrocellulose membrane. The membranes were blocked in 5% milk in PBS-Tween followed by incubation with mouse monoclonal primary antibodies anti-MRP1, anti-MRP2 (Monosan-Tebu, Le Perray en Yvelines, France) and anti-MRP3 (Chemicon international, Mundolsheim, France). Then, membranes were incubated with anti-mouse peroxydase-conjugated secondary antibodies (Sigma, St Quentin Fallavier, France) and ECL detection as described above.

#### Functional analysis of GST and MRP activities

Evaluation of GST activity was performed as follows. Cells treated or not with doxycycline were lysed in PBS pH 6.5 by three freezing (in liquid nitrogen) / defrosting (37°C) cycles and cytosols were recovered by centrifugation for 10 min at 4000 **g** and 4°C. The total GST activity was measured using CDNB as substrate (Depeille *et al*, 2004). Conjugation of reduced GSH to CDNB at 25°C and pH 6.5 was monitored spectrophotometrically at 340 nm.

The evaluation of MRP functional activity was assessed by studying [^3^H]-etoposide accumulation as described (Decleves *et al*, 2002). Cells, plated onto 12-microwell plates pretreated or not with doxycycline (0.2 *μ*g ml^− 1^, 24 h), were incubated for 1 h at 37°C with 5 *μ*M [^3^H]-etoposide in culture medium. After washing with PBS, cells were incubated at 37°C for 3 h in the absence (control) or presence of inhibitors (2 mM sulphinpyrazone, 30 *μ*M MK571, 30 *μ*M curcumin, 3.3 *μ*M ethacrynic acid). Then, cells were washed with ice-cold PBS to eliminate the extracellular tritiated drug and lysed with 500 *μ*l of 0.1 N NaOH. Intracellular [^3^H]-etoposide concentration was determined by *β*-scintillation counting and normalised to protein counting.

#### Cytotoxicity assays

The effect of anticancer agents on cell viability was assessed using the neutral red assay as previously described (Evrard *et al*, 1999; Depeille *et al*, 2004). Briefly, aliquots of cell suspension (10^4^ cells well^− 1^) were seeded in 96-well microtiter plates and incubated for 24 h at 37°C in the presence or absence of doxycycline (0.2 *μ*g ml^− 1^). Then, cells were exposed to mitomycin, chlorambucil, cisplatin, mitoxantrone and melphalan for 1 h at 37°C (150 *μ*l in fresh medium per well, eight wells per agent concentration). For doxorubicin, vincristine and etoposide, cells were exposed to drug for 4 h at 37°C in fresh medium supplemented with 1% fetal calf serum (FCS). When used, GST or MRP inhibitors were added 15 min before exposure to anticancer drugs. After 72 h incubation, cells were washed and a neutral red solution (33 *μ*g ml^− 1^) was added. After 3 h at 37°C, cells were washed and destained with glacial acetic acid (1%) – ethanol (50%) (v v^− 1^). Absorbances at 540 nm were measured using a microplate reader (Labsystems Multiscan MS). The effect of the drugs on cell survival was expressed as percentage of viability of treated-cells compared with control cells.

## RESULTS

### Characterization of cell lines

**Figure 1:**
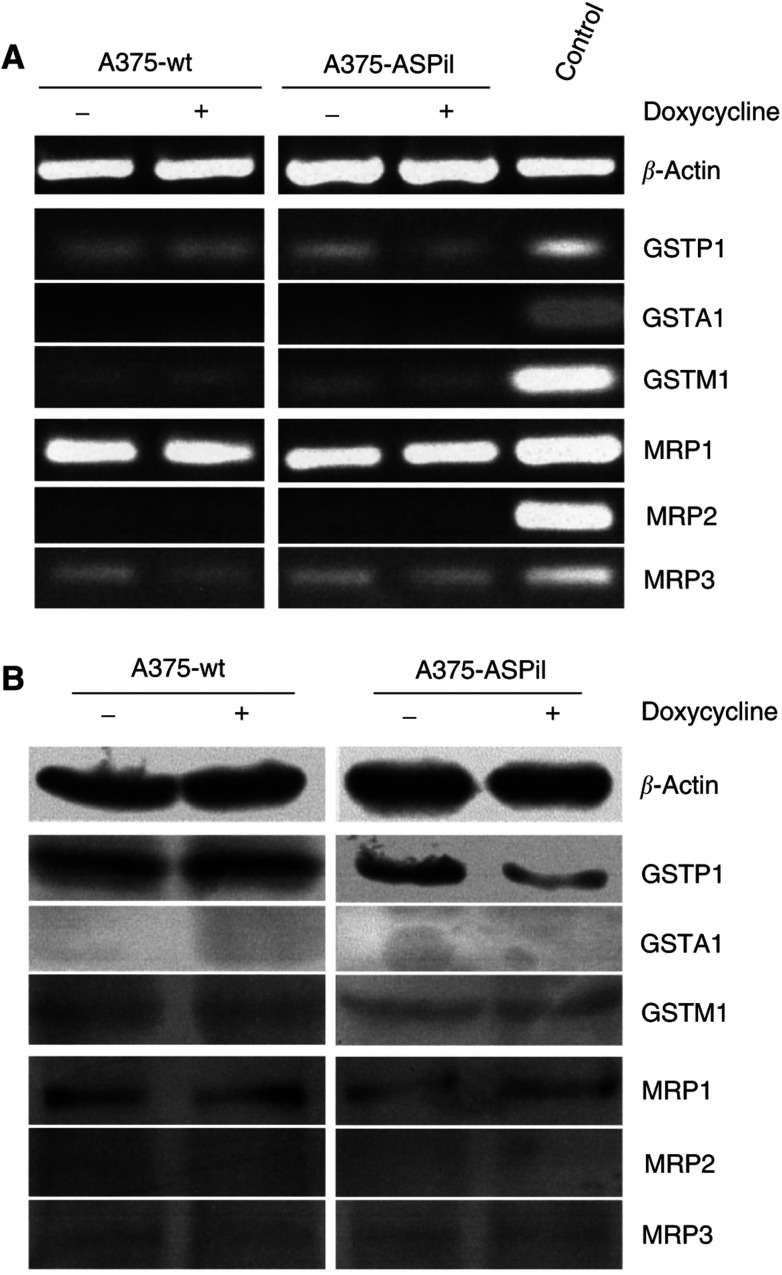
Expression of GSTs and MRPs in A375 cell lines. Levels of GSTs and MRPs mRNA (**A**) and (**B**) were determined by RT–PCR and Western blot, respectively, as described under ‘Materials and Methods’. Examined are cellular mRNA (4 ng each lane) and proteins (20 *μ*g and 100 *μ*g each lane for GSTs and MRPs, respectively) from parental cells (A375-wt) and cells transfected with pTet-On and ppTRE2-ASPi (A375-ASPi1). Experiments were made in the presence (+) or absence (−) of doxycycline (0.2 *μ*g ml^− 1^, 24 h). Positive controls were plasmids encoding GSTs (A1, M1, P1) and MRPs (1 and 2) or cDNA from Caco-2 cells for MRP3 for PCR experiments. *β*-Actin was used as a standardisation control in two detection experiments.

A375 MM cells (ATCC CRL1619) were chosen to assess the effects of the inhibition of GSTP1 expression on drug resistance. Reverse transcription polymerase chain reaction and Western blot experiments were designed to study the expression of GSTs and MRPs in A375 cells. As shown in Figure 1, high level of GST pi1 (GSTP1) and low level of GST mu1 (GSTM1) were detected in these cells, whereas GST alpha1 (GSTA1) was not found. Additionally MRP1 and MRP3, two ABC transporters involved in glutathione conjugate efflux (Keppler *et al*, 2000; Kruh and Belinsky, 2003) were identified (Figure 1). The expression level of MRP1 was higher than that of MRP3. In contrast MRP2, having overlapping substrate specificity with MRP1 (Smitherman *et al*, 2004), was not detected. Thus, A375 cells were a good model to study, by RNA AS strategy, the combined effects of GSTP1 and MRPs in MM chemoresistance.

As described in Materials and Methods, the controlled expression of GSTP1 AS RNA in A375 cells was obtained by using the Tet-On™ gene expression system. A375 cells were doubly transfected with two expression vectors: first with pTetOn containing the reverse rtTA, then with ppTRE-ASPi encoding human GSTP1 AS RNA, and double stable transfectants were selected for antibiotic resistance. The expression profiles of GSTs and MRPs in the double transfectant clones and in the parental A375-wt cells were similar (Figure 1).

**Figure 2:**
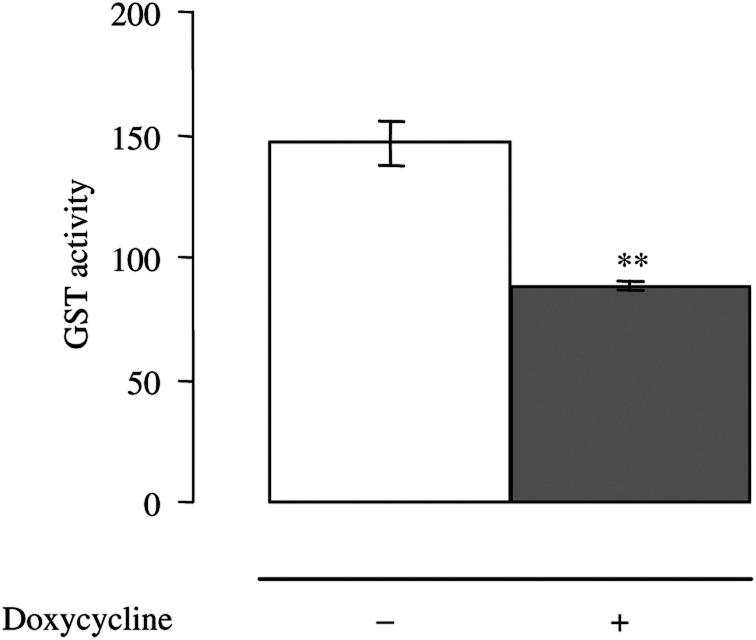
GST activities in A375-ASPi1 cells. Cytosolic proteins were extracted from A375-ASPi1 cells incubated in presence (+) or absence (−) of doxycycline (0.2 *μ*g ml^− 1^, 24 h) and assessed for their ability to conjugate CDNB to GSH as described in ‘Material and Methods’. Glutathione-S-transferase activities, expressed as nmol min^− 1^ CDNB conjugated with GSH per mg of cytosolic protein, are means±s.e.m. of at least three separate experiments. ^**^P<0.01 according to Student's *t*-test comparing values obtained in studied cells with those obtained in A375-ASPi1 cells in the absence of doxycycline.

Next, the clones were analysed for inhibition level of GSTP1 by AS RNA expressed under tetracycline control. The clone A375-ASPi1 displayed, in the presence of doxycycline (0.2 *μ*g ml^− 1^, 24 h), a specific 40% inhibition of GSTP1 mRNA and protein expression (Figure 1 and Table 1), and a significant decrease (about 40%) of total GST activity (Figure 2). Neither the expression of the other GSTs (A1 and M1) nor that of the MRPs was affected by doxycycline treatment in these cells (Figure 1).

**Figure 3:**
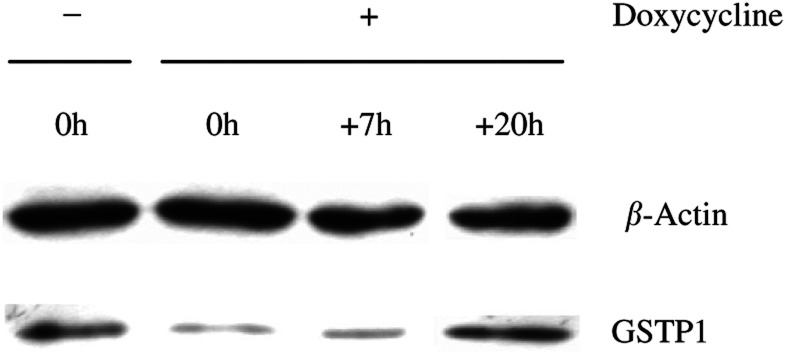
Duration of GSTP1 inhibition after doxycycline removal. Level of GSTP1 protein inhibition was determined by Western blot, as described under ‘Materials and Methods’. Examined are cytosolic proteins (20 *μ*g each lane) from A375-ASPi1 cultured in presence (+) or absence (−) of doxycycline (0.2 *μ*g ml^− 1^, 24 h). Lane 1, A375-ASPi1 were cultured in absence of doxycycline, corresponding to control cell line. Lanes 2, 3 and 4, A375-ASPi1 were cultured in presence of doxycycline during 24 h and lysed immediately, 7 and 20 h after doxycycline removal. *β*-Actin was used as a standardisation control in detection experiment.

To study the duration of the GSTP1 inhibition after doxycycline treatment, A375-ASPi1 cells were lysed either immediately or 7 to 20 h after removal of the tetracycline from the culture medium, and lysates were assayed for GSTP1 immunoreactivity. As shown in Figure 3, the 40% inhibition of GSTP1 level was still observed 7 h after removal of the doxycycline, whereas the expression level went back to basal value after a 20 h incubation period. Thus, the lowering of GSTP1 by AS RNA remained significant during the incubation time (at the most 4 h) of anticancer drugs in cytotoxicity assays. A375-ASPi1 cells presenting a tetracycline-controlled, significant, specific and lasting inhibition of GSTP1 expression were selected for further analysis.

### Effect of GSTP1 inhibition on the drug sensitivity of A375 cells

**Figure 4:**
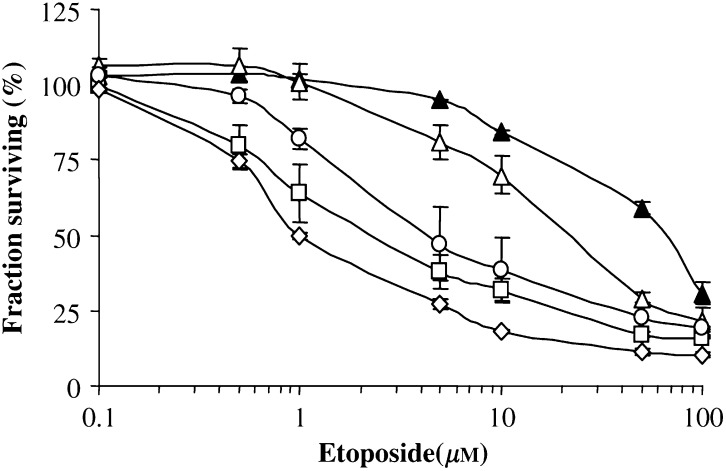
Effects of GSTP1 and MRP inhibition on the etoposide sensitivity of A375 cell lines. A375-ASPi1 cells were preincubated in presence (open symbols) or absence (closed symbols) of doxycycline (0.2 *μ*g ml^− 1^, 24 h) and then exposed for 4 h to etoposide alone (triangles) or with 30 *μ*M MK571 (square), 3.3 *μ*M EA (circle) or 30 *μ*M MK571 + 3.3 *μ*M EA (diamond) as described under ‘Materials and Methods’. Data are means±s.e.m. of three independent determinations.

We first studied the effects of GSTP1 inhibition by AS RNA on the drug sensitivity of A375 cell-ASPi1 cells with the use of the neutral red uptake assay. In our experimental conditions, the 24 h preincubation in the presence of 0.2 *μ*g ml^− 1^ doxycycline had no effect on cell viability. Anticancer drugs belonging to different therapeutic classes were tested: alkylating agents (chlorambucil, melphalan, mitomycin C, cisplatin), topoisomerase II inhibitors (doxorubicin, mitoxantrone and etoposide) and vinca alkaloid (vincristine). The corresponding data are summarised in Table 2. Inhibition of GSTP1 significantly decreased the resistance of A375-ASPi1 melanoma cells to etoposide (about three-fold) (Figure 4), but had no significant effect on relative resistance to the other agents tested. Moreover, the cytotoxicity profiles of parental A375-wt cells with or without doxycycline pretreatment were similar each other (data not shown), and were similar to that of A375-ASPi1cells in the absence of doxycycline (A375-ASPi1^(−)^) (Table 2).

To confirm the etoposide sensitising effect of the AS RNA-mediated reduction of GSTP1 expression in A375-ASPi1 cells, we tested whether inhibition of GST activity by the GST inhibitors curcumin (Harbottle *et al*, 2001) and ethacrynic acid (O'Dwyer *et al*, 1991) would also sensitise these cells. As shown in Figure 4 and Table 3, both inhibitors significantly amplified (about five-fold and two-fold, respectively) the AS RNA-mediated sensitisation of doxycycline-treated A375-ASPi1^(+)^ cells. The inhibitors had stronger (about 11-fold and 18-fold, respectively) sensitising effects in A375-ASPi1^(−)^ control cells expressing high GSTP1 level. The requirement of glutathione in the GSTP1-mediated protective effect was confirmed by using BSO, an inhibitor of glutathione synthesis (Bailey, 1998). In fact, glutathione depletion significantly increased the etoposide sensitivity of A375-ASPi1 cells (Table 2). None of the inhibitors used (curcumin, ethacrynic acid and BSO) had any effect on cell viability when used alone.

### Effect of MRP inhibitors on etoposide resistance of A375 cells

To investigate the involvement of endogenous MRPs in the etoposide resistance, cells were pretreated with MRP inhibitors before exposure to antineoplastic agents. As shown in Table 3 and Figure 4, sulfinpyrazone, an MRP1 selective inhibitor (Morrow *et al*, 1998) and MK571, an MRP1 and 3 inhibitor (Gekeler *et al*, 1995), significantly increased (about five-fold and 9.5-fold, respectively) the AS RNA-mediated etoposide sensitisation of doxycycline-treated A375-ASPi1^(+)^ cells; these compounds induced a stronger increase (up to 17-fold and 32-fold, respectively) of the etoposide sensitivity of A375 cells in the absence of doxycycline. None of the inhibitors used had any effect on cell viability when used alone.

These data, suggesting a combined implication of GSTP1 and MRP1 in the detoxification process of etoposide were confirmed by coincubating A375 cells in the presence of GSTP1 and MRP1 inhibitors (ethacrynic acid and MK571, respectively). The association of ethacrynic acid and MK571 significantly improved the etoposide sensitisation of A375-ASPi1 cells mediated by each inhibitor when used alone (about 3.5-fold and two-fold, respectively) (Table 3 and Figure 4).

### Requirement of GSTP1 and MRP1 in [^3^H]-etoposide accumulation

**Figure 5:**
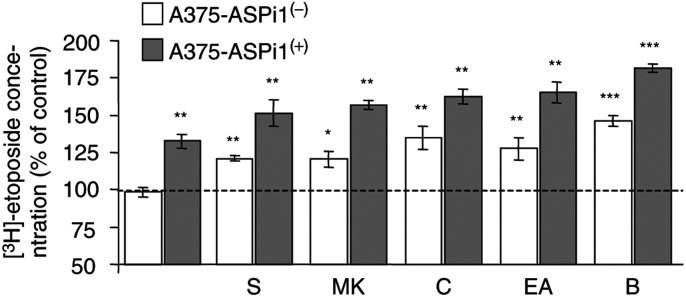
Functional activity of MRP1 in A375-ASPi1 cell lines. Cells were preincubated in presence (+) or in absence (−) of doxycycline (0.2 *μ*g ml^− 1^ 24 h). Then cells were incubated for 1 h at 37°C with 5 *μ*M [^3^H]-etoposide in culture medium. After washing with PBS, cells were incubated at 37°C for 3 h in the absence (control) or presence of inhibitors (2 mM sulphinpyrazone (S), 30 *μ*M MK571 (MK), 30 *μ*M curcumin (C), 3.3 *μ*M ethacrynic acid (EA)). For BSO 50 *μ*M (B), cells were preincubated 48 h before etoposide treatement. The intracellular [^3^H]-etoposide concentration was evaluated by *β*-scintillation counting as described in ‘Materials and Methods’. Data expressed as percentage of [^3^H]-etoposide concentration of control cells corresponding to A375-ASPi1 unexposed to inhibitors and to doxycycline are means±s.e.m. of three separate experiments. ^**^P<0.01; ^***^P<0.001 according to Student's *t*-test comparing values obtained with inhibitor in cells with those obtained without inhibitor.

To confirm the involvement of functional GSTP1 and MRP1 in the observed resistance of A375 melanoma cell to etoposide, we studied the effects of GSTP1 AS RNA expression, and of pharmacological inhibitors (curcumin, ethacrynic acid, BSO, sulfinpyrazone, MK571), on [^3^H]-etoposide accumulation. As shown in Figure 5, inhibition of GSTP1 expression by AS RNA significantly increased (about 35%) the accumulation of [^3^H]-etoposide in A375-ASPi1 cells. The involvement of functional GST in this effect was confirmed by using curcumin, ethacrynic acid and BSO, which significantly increased the accumulation of the tritiated compound in the cell lines. Moreover, MRP1 inhibitors (sulphinpyrazone, MK571) significantly increased the [^3^H]-etoposide accumulation in A375-ASPi1 cells.

## DISCUSSION

Malignant melanoma is a very chemoresistant tumour, which expresses, in 100% of individuals, high level of GSTP1 (Moral *et al*, 1997) and, in about 50% of melanoma specimens, high level of MRP1 (Schadendorf *et al*, 1995b). However, even if GSTP1 (Townsend and Tew, 2003) and MRP1 (Kruh and Belinsky, 2003) have been implicated, sometimes in a coordinated fashion (Harbottle *et al*, 2001), in the development of resistance toward chemotherapy agents, only a few studies have investigated their role in the drug resistance of MM and the results remained contradictory (Wang *et al*, 1989; Schadendorf *et al*, 1995a; Serrone and Hersey, 1999; Ichihashi and Kitajima, 2001). In this paper, we examined the effects of the inhibition of GSTP1 expression, by an inducible AS RNA, on drug sensitivity of human melanoma A375 cells in relation with endogenous MRP proteins.

A375 cells were chosen because their expression profile of GSTs and MRPs was similar to the expression profile of these glutathione-related detoxifying enzymes in individual tumours. In fact, as found in melanoma specimens (Schadendorf *et al*, 1995b; Moral *et al*, 1997), A375 cells expressed high levels of GSTP1 and MRP1, whereas very lower levels of GSTM1 and MRP3 were detected. The controlled inhibition by AS RNA of GSTP1 expression in A375 cells was obtained using the Tet-On™ gene expression system. A375 cells were successively transfected with two expression vectors encoding either reverse rtTA or human GSTP1 AS RNA, and selected for antibiotic resistance. The clone A375-ASPi1, which displayed a specific 40% inhibition of GSTP1 expression and a significant decrease of total GST activity in the presence of doxycycline (A375-ASPi1^(+)^) was selected for further analysis. In fact, even if the inhibition of GSTP1 expression in A375-ASPi1 cells by AS RNA was not complete, the observed percentage (about 40%) was comparable to that (about 50%) obtained in colon M7609 cells by using a retroviral expression vector to transfect GSTP1 AS cDNA (Ban *et al*, 1996). As suggested by Ban *et al* (1996), the degree of inhibition of gene expression by AS nucleotides depends on many factors including the levels of expression of the target gene as well as the amount of AS RNA transcribed. Furthermore, the 40% lowering of GSTP1 expression by AS RNA lasted for a time period (at least 7 h) greater than that (at the most 4 h) chosen for anticancer drugs treatment in cytotoxicity assays. Thus, A375-ASPi1 cells were a good model to study the effect of GSTP1 inhibition by AS RNA, in relation with endogenous MRPs, in MM chemoresistance. The cells expressing GSTP1 AS RNA in the presence of doxycycline were named A375-ASPi1^(+)^. The control cells used were parental A375-wt cells and the double transfectant ASPi1 clone in the absence of doxycycline (A375-ASPi1^(−)^).

A possible involvement of GSTP1 in etoposide resistance of human tumours was previously suggested by studies showing either an elevated GSTP1 in many cell lines selected in the drug (Tew, 1994) or a significantly influenced resistance by single transfection of GSTP1 (O'Brien *et al*, 2000). By using an AS approach, Ban *et al* (1996) observed a 2.1-fold increase of etoposide sensitivity after a 50% inhibition of GSTP1 expression. In A375 cells, a 40% reduction of GSTP1 expression level by inducible AS RNA was enough to induce a similar (about three-fold) increase of the etoposide sensitivity. This result, suggesting the involvement of GSTP1 in the resistance of MM to this topoisomerase II inhibitor, was confirmed by using pharmacological tools. The requirement of functional GSTs was shown by using the GST inhibitors curcumin and ethacrynic acid, which significantly reinforced the sensitising effect of GSTP1AS RNA in A375-ASPi1^(+)^ cells, and also strongly improved the etoposide sensitivity of A375-wt and A375-ASPi1^(−)^ control cells. The glutathione-dependency of the epipodophyllotoxin resistance of A375 cells was demonstrated by using BSO, an inhibitor of glutathione synthesis, which significantly increased the sensitivity of the cell lines to this agent. Taken together, these data strongly suggested a relationship between GSTP1 expression level and etoposide resistance of human melanoma. However, glutathione conjugates of etoposide have not been described and the molecular mechanism of the GSTP1-mediated protection remains unclear. A plausible protective role of GSTP1 could be, as suggested (O'Brien *et al*, 2000), a direct detoxification of quinone and semiquinone metabolites of etoposide, the latter forming conjugates with GSH, or of hydroxyl radicals generated from this metabolism. In favour of this hypothesis, it has been shown that these reactive forms could be produced by tyrosinases in melanoma cells and that toxicity of etoposide depended on presence of tyrosinase (Usui and Sinha, 1990). Alternatively, GSTP1 could act, as reported for inhibition of transcriptional activation by the peroxisomal proliferator-activated receptor gamma ligand, 15-deoxy-Delta(12,14)prostaglandin J(2) (Paumi *et al*, 2004), by sequestering etoposide in the cytosol away from its nuclear target.

Etoposide is a drug of the multidrug resistance phenotype (MDR) and the two MRP isoforms expressed in A375 cells, MRP1 and MRP3, were previously found to be implicated in etoposide resistance (Cole *et al*, 1994; Kool *et al*, 1999; Zeng *et al*, 1999; Zelcer *et al*, 2001). This finding was confirmed by using the MRP inhibitors sulfinpyrazone and MK571, which significantly increased the [^3^H]-etoposide accumulation and reduced the etoposide resistance of A375 cells. Considering that MRP3 on one hand is expressed at a very low level in A375 cells, on the other hand is only able to confer low levels of resistance to etoposide when ectopically expressed in cell lines (Zelcer *et al*, 2001), it is likely to assume that the MRP isoform mainly involved in A375 resistance to this natural product agent was MRP1. Furthermore, a significant increase of [^3^H]-etoposide accumulation in A375 cell lines was mediated by inhibition of GSTP1 expression (AS RNA), GST activity (curcumin and ethacrynic acid) or glutathione synthesis (BSO). The sensitising effect of the MRP inhibitors was stronger in cells expressing high GSTP1 level (A375-ASPi1^(−)^cells) than in cells expressing reduced GSTP1 level (A375-ASPi1^(+)^cells). Thus, as reported from nontumoral cells (O'Brien *et al*, 2000) the efficacy of the MRP1-mediated protection against etoposide was improved by the expression of functional GSTP1 in human melanoma A375 cells. A possible mechanism could be the implication of the glutathione metabolism in the export of etoposide from resistant cancer cells via MRP1. In fact, even if GS-conjugates have not been described, etoposide was shown to increase GSH export from cells expressing MRP1, suggesting cotransport with GSH (Rappa *et al*, 1997).

The sensitivity of the A375 cells to the other anticancer drugs tested in this study (doxorubicin, chlorambucil, melphalan, vincristine, cisplatin and mitoxantrone) was not affected by the 40% reduction of the GSTP1 expression level by AS RNA, indicating either that GSTP1 is not related to the resistance to these drugs, or that the degree of GSTPi1 inhibition is not sufficient to increase efficiently the drug sensitivity of the cells. This result is contradictory with previous Ban *et al*'s (1996) work, showing an increased sensitivity to etoposide, and also to doxorubicin, cisplatin and melphalan of colon M7609 after a similar half lowering of GSTPi1 intracellular level by AS transfection. However, it is in accordance with other studies reporting the lack of correlation between GSTP1 expression level and the sensitivity of different cancer cell lines to the drugs mentioned above (Fairchild *et al*, 1990; Morrow *et al*, 1998; Wang *et al*, 1999). Our explanation for this discrepancy is that the detoxification processes seems to be highly specific on one hand for the drug / GST isozyme / MRP isozyme combination, on the other hand for the cell or tissue concerned. This hypothesis is supported by recent investigations suggesting that the effects of GSTs and / or MRPs expression on anticancer drug toxicity depend on the cell type (Morrow *et al*, 1998; Harbottle *et al*, 2001; Depeille *et al*, 2004).

In conclusion, we report a combined action of GSTP1 and MRP1 in the protection of A375 melanoma cells from etoposide effect. These data provide rationale for studying the relationship between the expression levels of GSTP1 and MRP1 in MM tumours and the clinical outcome of patients treated with etoposide. In contrast, the absence of effect of GSTP1 inhibition against the other agents tested (doxorubicin, chlorambucil, melphalan, vincristine, cisplatin and mitoxantrone) strongly supports the finding that the action of detoxification processes is very dependent on the tissue concerned.

## Figures and Tables

**Table 1:** Level of GSTP1 mRNA in A375-ASPi1 cells

**Cell lines**	**GSTP1 mRNA normalized ratios**
CAL1-*μ*1	1.0
A375-ASPi1^(−)^	1.1±0.1
A375-ASPi1^(+)^	0.6±0.04

Exponents ^(+)^ and ^(−)^ indicate that A375-ASPi1 cells were pretreated in the presence or absence of doxycycline, respectively. CAL1-*μ*1 cell line was used as calibrator. GSTP1 mRNA normalized ratios were obtained as described in ‘Material and Methods’. Data are means from three separate experiments.

**Table 2:** Effects of the inhibition of GSTP1 expression by antisense RNA on cellular sensitivities to anticancer drugs

**Anticancer drugs**	**Fold sensitisation**
Cisplatin	0.83
Chlorambucil	1.11
Melphalan	0.9
Etoposide	3.33^***^
Mitoxantrone	0.83
Doxorubicine	1.11
Vincristine	1

Fold sensitisation=IC_50_ of A375-ASPi1^(−)^ / IC_50_ of A375-ASPi1^(+)^. Exponents ^(+)^ and ^(−)^ indicate that cells were pretreated in the presence or absence of doxycycline, respectively. Data are means from at least three separate experiments. ^***^P<0.001 (Student's *t*-test).

**Table 3:** Effects of inhibitors of MRP (sulphinpyrazone, MK571), GST (curcumin, ethacrynic acid) and GSH (BSO) on cellular sensitivities to etoposide

	**Fold sensitisation to etoposide**		
**Inhibitor**	**A375-wt^(−)^**	**A375-ASPi1^(−)^**	**A375-ASPi1^(+)^**
Ethacrynic acid	19^***^	18.4^***^	5^*^
Curcumin	11^***^	11.4^***^	2.4^*^
BSO	21.3^***^	22.6^***^	6.8^**^
Sulfinpyrazone	18.1^***^	17^***^	5^*^
MK571	32^***^	33.3^***^	9.5^**^
Ethacrynic acid and MK571	ND	70^***^	17.5^***^

Fold sensitisation=IC_50_ of etoposide in the absence of inhibitor / IC_50_ of etoposide in the presence of inhibitor. Ethacrynic acid, curcumin, BSO, sulphinpyrazone and MK571 were used at 3.3 *μ*M, 30 *μ*M, 50 *μ*M, 2 mM and 30 *μ*M, respectively. Exponents ^(+)^ and ^(−)^ indicate that cells were pretreated in the presence or absence of doxycycline, respectively. ND: not determined. Data are means from at least three separate experiments. ^*^P<0.05, ^**^P<0.01, ^***^P<0.001 (Student's *t*-test).
